# Evaluating AVAHAN’s design, implementation and impact: lessons learned for the HIV Prevention Community

**DOI:** 10.1186/1471-2458-11-S6-S16

**Published:** 2011-12-29

**Authors:** Marie Laga, Bea Vuylsteke

**Affiliations:** 1 ITM-HIV-AIDS- Center, Department of Public Health, Institute of Tropical Medicine, Nationalestraat 155, 2000 Antwerpen, Belgium

## 

AVAHAN, the world’s largest HIV prevention programme to date, needs no introduction to the international prevention community. Since its launch in 2003, AVAHAN and partners published over 100 papers in the peer reviewed literature in addition to the many presentations at AIDS conferences.

Time for some reflections on how AVAHAN was designed, implemented and evaluated. What are the lessons learned for planning and managing HIV prevention programmes?

## The programme design: did they do the right thing?

AVAHAN ‘s choice to focus on prevention in early 2000 was timely and strategically right. India was experiencing a rapidly growing epidemic, concentrated in female sex workers and their clients, high risk MSM and transgender, and injecting drug users in the North east [1]. The decision to focus on key populations only was controversial at the time, because of the ongoing debate whether India’s epidemic was becoming a generalized epidemic. Moreover, around 2003 -5 the world wide efforts and resources went almost entirely to antiretroviral scale up, making it hard to put prevention on the agenda. Looking at it now, the strict focus on targeted prevention was certainly one of the critical steps towards success. Based on “understanding the Indian epidemic” the response was tailored to the key populations, at highest risk for acquiring and or transmitting HIV and in the highest prevalence states. The populations were mapped, as well as the already ongoing programmes implemented by the government and other partners, and AVAHAN opted to cover the large gaps.

The core programme components offered to the key populations were evidence based [2,3], and adapted to the specific needs of the populations in India. They included: peer-led outreach and behaviour change communication; services for STI testing and care and condom promotion and distribution; and harm reduction for injecting drug users. Community mobilization approaches, cutting across all programmes, were essential to AVAHAN’s programme design. The positive experiences from the Indian Sonagachi sex worker programme were inspiring [4]. Dr Jana, a key leader from Sonagachi, advised AVAHAN in the design phase. But the decision to include community mobilization was equally driven by pragmatism and common sense. The senior staff of AVAHAN recognized that creating an enabling environment when working with highly stigmatized and marginalized populations was essential for programme improvement. Police harassment or violence against women, which could impede uptake of services or use of condoms, were addressed. It also became clear that the populations targeted had more pressing needs than HIV prevention, which could be met. Those included, for example, a cash transfer system with cell phones for the sex workers, a safe haven drop in center for transgenders or shower facilities for the truck drivers. Support to “self-organization” of the communities was seen as a way to enhance community buy-in eventually resulting in a more sustained response. Was this evidence-based policy making? Strictly speaking no, because there is little empirical evidence that such structural interventions reduce HIV transmission [5]. And this will likely remain so because of the complexity of obtaining this evidence with the gold standard methods. Was it the right decision? Certainly yes, because it made sense, from a programme logic perspective. It was sound prevention planning, taking into account the dynamics of the local epidemic, the populations in their context, the evidence base and the “lessons learned from other programmes”. And the intention was “learn as we go”. The only critical note may be that AVAHAN’s evaluation design did not include the community mobilization from the start. More reflection on how those processes could be defined, quantified, and evaluated in a prospective way would have been helpful, and allow to make a stronger case about relative contribution of the community mobilization components to the overall impact.

## Programme implementation: did they do it right?

Undoubtedly, AVAHAN is an example of rapid scale up and implementation success [6]. It reached its coverage targets as planned, ultimately resulting in good outcomes and impact. As an illustration, together with 9 lead partners, 134 grass roots organizations were supported to train over 7000 peer educators to reach 280.000 sex workers in less than 3 years. The question is therefore not *whether* rapid scale up happened, but rather *how* that happened.

Most of the senior management team of AVAHAN came from the private sector, and had strong management experience. It has been said numerous times, the “ business approach” is without any doubt a key ingredient for the rapid scale up, good programme uptake or consistent commodity supply [7]. No matter how sound the programme planning and design, if no attention is paid to the management details, the result will be programme failure. Relevant lead partners and grass roots NGO’s were carefully selected. A sense of urgency was introduced from the beginning. The option was scaled design, not scaling up from a pilot project, and the approach was clearly result oriented. An extensive monitoring system was set up, tracking all programme components including at the lowest community level. And last but not least, the data were used for regular programme review and adjustments. Not just for the donor or for presentation in international conferences. There were also attempts to measure programme quality, even if that component is so far the least developed.

The lesson learned here is that prevention programmes can and should benefit more from private sector experience. Key business management principles and public health programming may seem two different worlds with little in common. AVAHAN with its atypical senior management staff, has been a good illustration that business approaches can greatly enhance public health programmes [7]. A better mix of managers and “health problem“ experts could advance the HIV prevention agenda as well as other public health challenges.

## Programme evaluation: did it work?

Recently, Ng and colleagues estimated that AVAHAN had averted 100.178 new HIV infections in a 5 years period between 2003-08. [8]. This number can be critized for the way it was obtained ( methods and data used), and the high levels of uncertainty [9], but it is encouraging to see the impact finally quantified. More than 100.000 human beings saved from suffering, lifelong treatment or premature death. It helps to convince the critics about the effectiveness of prevention in general. And it makes a strong case that large scale prevention programmes, offering basic programme components to sex workers, without the new magic- bullets prevention tools such as male circumcision and ART as prevention, can have a significant effect at the population level.

But those estimates are just the cherry on the cake of an extensive evaluation effort that accompanied the implementation of AVAHAN [10]. The prospective evaluation design included at least 2 consecutive surveys, integrated behavior and biologic assessments (IBBA) in all targeted populations, several district population based surveys, monitoring of programme implementation data and intervention uptake, as well as cost estimates. And investment with modeling was extensive. After summarizing and triangulating the overwhelming amount of data, the effectiveness of AVAHAN is truly convincing. Encouraging trends in condom uptake and declines in STI and HIV rates in key populations are observed almost consistently. The triangulation of programme scale up data, with outcome data and trends if HIV in key populations and in pregnant women, allow for a strong plausible case that AVAHAN has had impact. It contributed significantly to the overall decline of HIV among sex workers, and their clients, and subsequently in the general population in some states. The impact is less convincing in the Northern states, where injecting drug use is the main mode of transmission, partly due to less data available so far to be used in the triangulation and modelling. There is a need to build a more coherent case of evidence of harm reduction impact, using as much quantitative and qualitative data.

## Evaluation design: issues and missed opportunities?

Despite careful planning and sufficient funding, the prospective evaluation design has been criticized for not having implemented the programme in a randomized phase fashion [8,11]. It is clear that such a large scale programme could not have been implemented easily in a randomized way, and ethical debates within India prevented it. But AVAHAN was right not to adapt and complicate their implementation only to suit the needs of a “probability evaluation design”. Requesting large scale programs to implement in a randomized way so that impact can be assessed, is worrisome [12]. Especially if this leads to slower scale up or more narrow programme packages, leaving out the context specific community responses.

Another criticism was the lack of a real baseline. The first IBBA round was done when the programmes were already well in place. While regrettable, the many logistic constraints, not the least of ethical clearances, prevented the assessments from being completed in time to be true baselines. But a more fundamental question can be asked. Is it ethical and possible to do an anonymous survey including blood drawing among a highly marginalized population of sex workers, before gaining their confidence and putting any services in place? The need for rigorous evaluation designs is recognized, but not at a cost of invasive strategies that can cause harm of discrimination or stigmatisation.

Have there been no missed opportunities with regard to impact measurement in AVAHAN? More real life evaluation at the programme and clinic sites would have been useful. This approach could have produced baseline data on critical parameters such as condom use among first attenders. Even if those absolute estimates are biased, they are useful for interpretation of trends, and provide arguments to build a plausible case of possible programme impact. Also more qualitative data could have strengthened the learning dimension, on why impact occurred where. And thirdly, the evaluation of the community mobilization approaches should have been planned in advance, as was done for the rest of the programme. This is recognized now, and attempts are made to try to evaluate the contribution of the community mobilization to the impact in a retrospective way, using theoretical frameworks and modeling.

## How much evaluation is enough?

Looking at the huge amount of data that have been collected, and still is being collected and analyzed to evaluate the different aspects of the programme, the question arises whether the effort and resources for evaluation are still in balance with those for implementing more programmes? The answer depends on whom you ask the question: the funder, the government of India, the stakeholders and beneficiaries of the programmes or the International Prevention Community.

The Bill and Melinda Gates foundation, as funder should be very satisfied with the rapid scale up, programme fidelity and uptake of this programme, including large scale behavior changes. And even more about the growing evidence that Avahan contributed significantly to the declining trend of the Indian HIV epidemic, in addition to non-HIV outcomes, such as reduction in violence against women. It will be essential to digest this wealth of evaluation data into a few key lessons and main messages for the government of India and the national and regional AIDS control program. Now that Avahan is in the process of being transitioned to the Government, justification to continue with targeted programs and community approaches while keeping the momentum on Prevention, will be essential. In addition, lessons learned from regional variations in programme success and quality should be shared. The communities as the beneficiaries of the programme deserve to be informed about the main evaluation results, because they were not only part of the problem, but also part of the solution.

For the international HIV prevention evidence base, AVAHAN’s extensive evaluation efforts are crucial. For the first time, it has been shown, with a prospective design, that a large scale targeted HIV prevention programme, including standardized programme packages reinforced with community mobilization approaches, is possible and works. It leads to improved outcomes and prevents new infections in the population most at risk, ultimately affecting the epidemic as whole. A summary paper, or rather a book, analyzing carefully the key pieces of the evidence, is urgently needed.

The prevention world is currently facing new dilemmas such as antiretroviral treatment for prevention, when large numbers of AIDS patients worldwide are still dying because of lack of access. The lessons learned from AVAHAN may bring some realism to this debate. Prevention “ back to the basics” can prevent many HIV infections. In areas of Africa or Asia, where scale and coverage of interventions for most at risk populations remains the greatest challenge, this could be a way to start!
